# The Effect of Heat Shock Protein 90 Inhibitor on Pain in Cancer Patients: A Systematic Review and Meta-Analysis

**DOI:** 10.3390/medicina57010005

**Published:** 2020-12-23

**Authors:** Victoria N. Miles, Roma K. Patel, Amanda G. Smith, Ryan P. McCall, Jun Wu, Wei Lei

**Affiliations:** Department of Pharmaceutical and Administrative Sciences, Presbyterian College School of Pharmacy, Clinton, SC 29325, USA; vnmiles@presby.edu (V.N.M.); rkpatel@presby.edu (R.K.P.); agsmith@presby.edu (A.G.S.); rpmccall@presby.edu (R.P.M.); jwu@presby.edu (J.W.)

**Keywords:** heat shock protein 90 inhibitor, pain, cancer, clinical trial, meta-analysis

## Abstract

*Background and objectives*: Heat shock protein 90 (Hsp90) is a molecular chaperone that plays an essential role in tumor growth. Numerous Hsp90 inhibitors have been discovered and tested in preclinical and clinical trials. Recently, several preclinical studies have demonstrated that Hsp90 inhibitors could modulate pain sensitization. However, no studies have evaluated the impact of Hsp90 inhibitors on pain in the patients. This study aims to summarize the pain events reported in clinical trials assessing Hsp90 inhibitors and to determine the effect of Hsp90 inhibitors on pain in patients. *Materials and Methods*: We searched PubMed, EBSCOhost, and clinicaltrials.gov for Hsp90 inhibitor clinical trials. The pain-related adverse events were summarized. Meta-analysis was performed using the data reported in randomized controlled trials. *Results*: We identified 90 clinical trials that reported pain as an adverse effect, including 5 randomized controlled trials. The most common types of pain reported in all trials included headache, abdominal pain, and back pain. The meta-analysis showed that Hsp90 inhibitors increased the risk of abdominal pain significantly and appeared to increase the risk for back pain. *Conclusions*: In conclusion, Hsp90 inhibitor treatment could potentially increase the risk of pain. However, the meta-analysis demonstrated only moderate evidence for the connection between Hsp90 inhibitor and pain.

## 1. Introduction

Heat shock protein 90 (Hsp90) is a molecular chaperone protein found in vertebrates, invertebrates, plants, protists, and even bacteria [1]. Hsp90 assists in the process of maturation and folding of client proteins [2], stabilization and activation of kinases [3,4], cross-linking of actin filaments [5], and condensation of chromatin in the nucleus [6]. Elevated levels of Hsp90 have been found in the process of tumor growth [7]. The involvement of Hsp90 in uncontrolled growth associated with cancer has led to the investigation of Hsp90 inhibitors as potential anti-cancer therapies. The safety and efficacy of various Hsp90 inhibitors have been tested in phase I, phase II, and phase III clinical trials, both as monotherapy and in combination with other anti-cancer agents [8]. Although they showed some promise in cancer treatment, the first generation of Hsp90 inhibitors, such as 17-(Allylamino)-17-demethoxygeldanamycin (17-AAG) and 17-dimethylaminoethylamino- 17-demethoxygeldanamycin (17-DMAG) have demonstrated adverse events such as hepatotoxicity, nausea, vomiting, diarrhea, and hypersensitivity [9]. In an effort to reduce the adverse event profile of these drugs as well as improve anti-cancer efficacy, the second generation of Hsp90 inhibitors has been developed. The second generation of Hsp90 inhibitors, including ganetespib, have shown significant improvement in the performance of cancer therapy. However, they may still cause neutropenia, leukopenia, anemia, pain, and diarrhea [10].

Pain is defined as an unpleasant sensory and emotional experience associated with actual or potential tissue damage [11]. Pain is a serious symptom of cancer patients and leads to significant economic impact and a drastic decrease in quality of life [12]. To date, approximately 75% of cancer patients experience pain [13], and one-third of cancer survivors continue to experience chronic pain throughout their lifetime [14]. Pain can be elicited during the process of cancer and immune responses or during cancer treatment [15]. The activation of immune cells promotes the release of cytokines, neuropeptides, and neurotransmitters, which result in the pain sensitization [16]. Cancer pain can also be associated with the treatment for cancer, such as peripheral neuropathy induced by chemotherapy [17].

Preclinical studies have shown evidence of a relationship between Hsp90 and pain. When the Hsp90 inhibitor geldanamycin was administered subcutaneously at varying doses in rat models, a reduction in mechanical allodynia and a higher threshold to pain was observed [18]. Systemic administration of 17-AAG and intrathecal administration of 17-DMAG in these models produced similar results, further demonstrating that Hsp90 could impact pain sensitization [19]. Treatment with the novel Hsp90 C-terminal inhibitor, KU-32, slowed the progression of diabetic neuropathy through the motor and sensory nerve conduction velocity in mouse models [20]. Reversal of disease progression markers, including motor and sensory nerve conduction velocity, mechanical and thermal sensitivity, and intra-epidermal nerve fiber density, were observed [21]. The findings from these preclinical studies indicate that Hsp90 could regulate pain signaling, particularly in neuropathic pain. However, the relationship between Hsp90 inhibitors and pain has not been directly investigated in clinical trials, which means that there is little clinical evidence to establish the association between Hsp90 and pain. This study aimed to summarize the pain-related adverse effects reported in the clinical trials testing various Hsp90 inhibitors. Furthermore, we performed meta-analysis to determine the impact of Hsp90 inhibitors on pain in cancer patients.

## 2. Materials and Methods

### 2.1. Search Strategy

We conducted searches of EBSCOhost, PubMed, and clinicaltrials.gov during the months of May and June 2020 to identify all clinical trials investigating inhibitors of HSP90. The following search terms were used: “HSP90 inhibitor”, “17-AAG”, “17-DMAG”, “AT-13387”, “AUY922”, “STA-9090”, “SNX-5422”, “HSP990”, “IPI-504”, and “BIIB021”. Trials were collected up to 1 June 2020. Additionally, we searched conference proceedings and screened the reference list of relevant papers.

### 2.2. Inclusion and Exclusion Criteria

Titles and abstracts were screened to exclude animal studies, reviews, and observational studies. Four reviewers (Miles, Patel, Smith, and McCall) independently evaluated studies for eligibility and eliminated duplicated publications found among the three databases. The characteristics of the eligible publications were extracted by the reviewers, including the year of publication, sample size, treatment, regimen, control group, and indications. The completed data forms were checked by two reviewers (Lei and Wu).

Clinical trials, both randomized and non-randomized, investigating any HSP90 inhibitor at any dose with published results were included in the analysis. Trials that were ongoing or withdrawn/terminated before completion were excluded from the analysis. Further, completed studies with no results published or no pain-related adverse events reported were also excluded.

The systemic review was conducted for eligible trials without control groups to summarize the incidence of pain-related adverse events. Meta-analysis was performed for randomized controlled trials to evaluate the effect of Hsp90 inhibitors on selected pain events.

### 2.3. Outcomes

The primary outcomes were pain-related adverse events reported by three or more randomized-controlled trials, including arthralgia, back pain, myalgia, pain in the extremities, headache, peripheral neuropathy, and abdominal pain. The odds ratio (OR) and confidence interval (CI) were estimated for each type of pain event in each study to reflect the effect of the treatment on the pain-related events. The secondary outcomes were other pain-related adverse events reported by clinical trials with or without control groups. The incidences of the pain-related events in each study were described.

### 2.4. Statistical Analysis

The I^2^ statistic was calculated to test heterogeneity of the studies included for primary outcome measures. The I^2^ statistic reflects the percentage of total variation among the studies that is attributable to heterogeneity [22]. The ORs were pooled using a fixed-effect model (I^2^ < 50%) for back pain, pain in the extremities, headache, peripheral neuropathy, and abdominal pain, and using a random-effect model (I^2^ ≥ 50%) for arthralgia and myalgia. The CIs were computed at the 95% level. Forest plots described the combined and individual ORs and 95% CIs. All analyses were performed using SAS 9.4.

## 3. Results

### 3.1. Search Results

**Figure 1:**
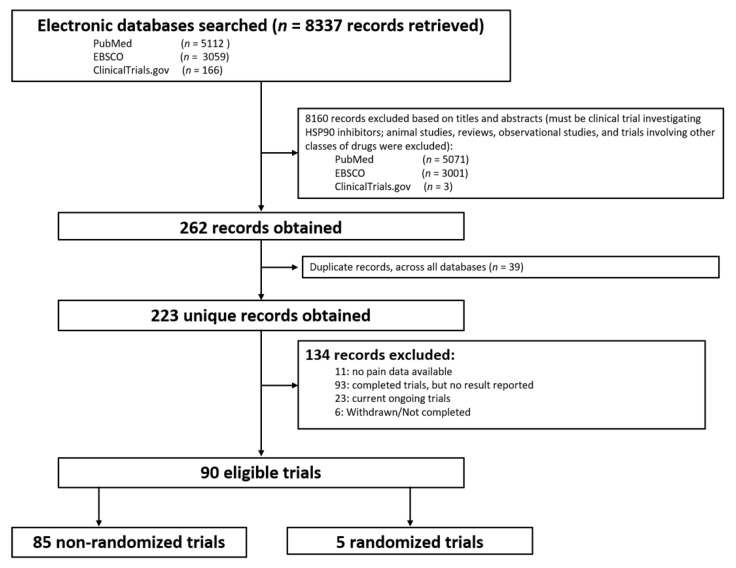
Flow diagram of identification of eligible trials.

The database search yielded 8337 potential studies. After removing preclinical studies, reviews, observational studies, and duplications, 226 studies remained. A total of 134 studies were excluded due to no data or no pain-related data reported, or incomplete studies. Finally, a total of 90 clinical trials were included that reported pain-related events (Figure 1 and Supplemental Table S1), including 85 phase I or II trials without control groups and 5 randomized controlled phase II or III clinical trials.

### 3.2. Pain-Related Adverse Events Reported by Clinical Trials

The pain events reported by clinical trials with or without a control group are listed in Supplemental Table S1. As summarized in Table 1, ganetespib, 17-AAG, and luminespib (AUY922) have been tested in numerous trials in patients with different types of cancer. The most common pain reported by patients included headache (15.1%), abdominal pain (10.8%), back pain (8.1%), peripheral neuropathy (6.4%), and myalgia (6.4%).

### 3.3. Meta-Analyses

Table 2 displays the characteristics of the five randomized controlled trials included for analysis. All reported treatments were combinations of Hsp90 inhibitors and agents used in the control group. The control groups in the five trials were docetaxel, paclitaxel, or crizotinib. Four trials reported back pain and headache events and three trials reported arthralgia, myalgia, pain in the extremities, peripheral neuropathy, and abdominal pain, respectively. The combined four studies yielded a population of 1112 participants for back pain and 996 participants for headache. Three trials included participants with non-small cell lung cancer.

**Figure 2:**
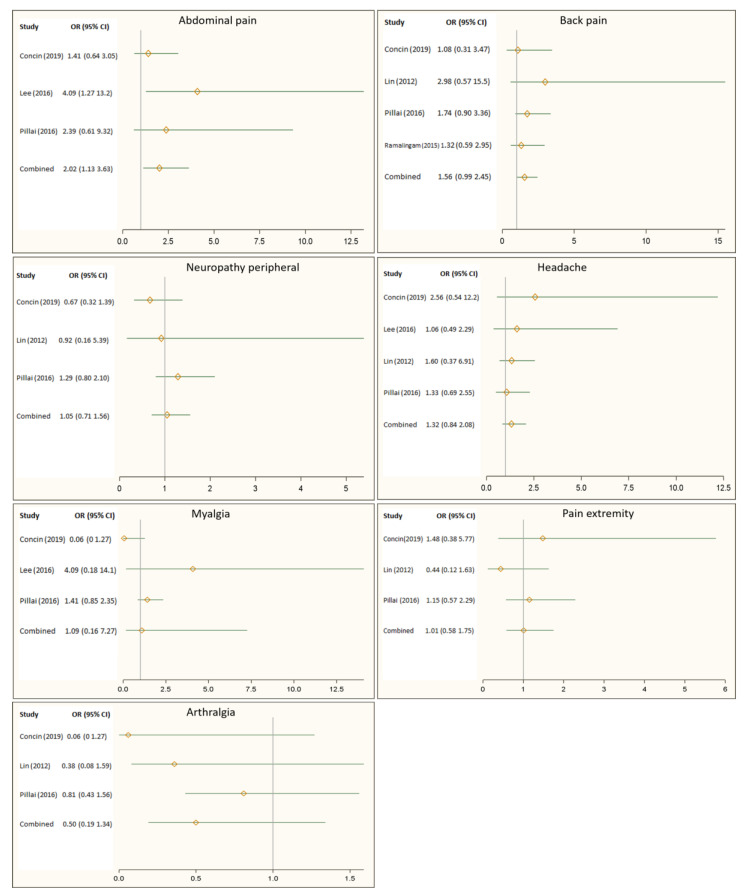
Impact of Heat shock protein 90 (Hsp90) inhibitors on back pain, headache, peripheral neuropathy, abdominal pain, myalgia, pain in the extremities, and arthralgia.

Figure 2 illustrates the effect of the Hsp90 inhibitors on pain-related adverse events reported by randomized controlled trials. Overall, the Hsp90 inhibitors appeared to increase the risk of back pain by 56%, headache by 32%, and myalgia by 52%. However, these increases in risk did not show statistical significance. Figure 2 also showed that patients receiving Hsp90 inhibitors were 2 times more likely to report abdominal pain than those in the control group (OR: 2.02, 95% CI: 1.13–3.63).

## 4. Discussion

This study demonstrated that patients in randomized control trials receiving Hsp90 inhibitors had a statistically significant increase in reported abdominal pain versus control. Back pain and headache showed a trend towards increased incidence. Peripheral neuropathy, myalgia, pain in the extremities, and arthralgia were also commonly reported in the patients treated with Hsp90 inhibitors. This study is the first to directly compare the effect of treatment with Hsp90 inhibitors versus controls on types of pain reported as adverse events.

Several Hsp90 inhibitors have been evaluated for their impact on tumor progression. This first generation of Hsp90 inhibitors includes 17-AAG, 17-DMAG, and IPI-504 [27]. They bind to the ATP-binding site at the N-terminal domain of Hsp90, resulting in the degradation of most client proteins [3]. Those Hsp90 inhibitors showed activities to reduce the tumor growth in the clinical trials. Meanwhile, patients treated with first generation Hsp90 inhibitors were subject to severe hepatic and ocular toxicity [28]. In an effort to diminish these adverse effects, the second generation of Hsp90 inhibitors has been developed. This new generation targets the C-terminal domain of Hsp90, such as onalespib (AT13387), ganetespib (STA-9090), luminespib (AUY922), SNX-5422, BIIB-021, PU-H71, XL-888, HSP990, KW-2478, Debio-0932, KU-32, TAS-116, and dacinostat (LAQ-824). These drugs have successfully decreased the incidence of hepatic and ocular toxicity [27]. However, some severe pain-related adverse effects have been reported in trials testing either the first or second generations of Hsp90 inhibitors. The current process of discovering Hsp90 inhibitors is targeting specific isoforms or co-chaperones of Hsp90, which might be helpful for eliminating such side effects shown in the patients.

Hsp90 has been demonstrated to be involved in the sensitization of pain. Levels of Hsp90 messenger RNA were increased in dorsal root ganglia in an arthritis rat model, and administration of 17-DMAG reduced monoarthritis-induced mechanical allodynia [18]. Similarly, chronic constriction injury-induced allodynia was suppressed with systemic administration of 17-AAG and intrathecal administration of 17-DMAG in mice [19]. KU-32 is a novobiocin analogue Hsp90 inhibitor being investigated for the treatment of diabetic neuropathy. In preclinical mouse studies, treatment with this Hsp90 inhibitor demonstrated slowed disease progression through the motor and sensory nerve conduction velocity [20]. Additionally, the administration of KU-32 reversed disease progression markers, including motor and sensory nerve conduction velocity, mechanical and thermal sensitivity, and intra-epidermal nerve fiber density [20,21]. These preclinical findings suggest that Hsp90 inhibitors reduce the sensitization of different types of pain, such as neuropathic pain. In this study, the meta-analysis found that treatment with Hsp90 inhibitors increased abdominal pain and back pain (in trend), but showed no significant association between treatment with Hsp90 inhibitors and other types of pain in clinical trials. These differences may be due to the pain investigated in the preclinical models that were not cancer related. Another reason may be related to the activity of Hsp90 inhibitors on pain drugs, such as opioids. Previous studies showed that inhibition of Hsp90 in the mouse brain could diminish the opioid-induced signaling and behavior [29,30]. The increased risk of pain by Hsp90 inhibitors could relate to the attenuation of pain management in the patients. Unfortunately, none of the randomized controlled trials reported how the pain was managed in those patients.

Several limitations should be considered when the meta-analysis results are interpreted. First, the number of published studies that were eligible for analysis was relatively small. The majority of the clinical trials were phase I or II without control groups. However, heterogeneity was tested, and variations were controlled when the studies were combined for analysis. Second, indications and control groups in each study varied, which increased the level of heterogeneity of the publications. The random-effect model was applied to address the heterogeneity in the analysis. Due to the low incidence of arthralgia and myalgia reported in some studies, the wide 95% CIs could bias the results. Third, based on available information reported in clinical trials, the pain events were classified by anatomical area. Since the different types of underlying malignancy could affect the type of pain, assessing the pain associated with the Hsp90 inhibitor by underlying malignancy would enhance the knowledge of pain-related side effects in Hsp90 inhibitor development. In addition, pain outcomes were reported by patients in the trials. Whether patients were informed of pain as a potential side effect in the trial could influence patients’ perceptions and confound pain outcome measures. However, such information was not available in the publications. Finally, some trials were terminated without results. Thus, publication bias could influence the conclusion.

## 5. Conclusions

In conclusion, this systematic review and meta-analysis found moderate evidence indicating that treatment with Hsp90 inhibitors increases the risk of abdominal pain, but not any other types of pain. Pain-related adverse events should be collected in future clinical trials testing Hsp90 inhibitors in cancer patients. More data from clinical trials are needed to further confirm the link between Hsp90 and pain.

## Figures and Tables

**Table 1:** Summary of pain in Hsp90 inhibitor clinical trials.

Pain	17-AAG (*n* = 689)	Ganetespib (STA-9090) (*n* = 994)	AUY922 (*n* = 420)	17-DMAG (*n* = 154)	IPI-504 (*n* = 178)	Other Hsp90 Inhibitors * (*n* = 1022)	Total (*n* = 2841)
Pain	11 (1.60%)	43 (4.33%)	9 (2.14%)	3 (1.95%)	- ^#^	60 (5.87%)	126 (4.4%)
Back pain	47 (6.82%)	107 (10.76%)	34 (8.10%)	7 (4.55%)	30 (16.85%)	5 (0.49%)	230 (8.1%)
Headache	76 (11.03%)	85 (8.55%)	82 (19.52%)	14 (9.09%)	52 (29.21)	121 (11.11%)	430 (15.1%)
Pain in extremities	9 (1.31%)	50 (5.03%)	10 (2.38%)	-	4 (2.25%)	1 (0.10%)	74 (2.6%)
Arthralgia	24 (3.48%)	38 (3.82%)	2 (0.48%)	19 (12.34%)	41 (23.03%)	3 (0.29%)	127 (4.5%)
Bone pain	16 (2.32%)	24 (2.41%)	4 (0.95%)	-	-	-	44 (1.5%)
Myalgia **	49 (7.11%)	43 (4.33%)	24 (5.71%)	12 (7.79%)	37 (20.79%)	17 (1.56%)	182 (6.4%)
Abdominal pain ***	38 (5.52%)	114 (11.47%)	59 (14.05%)	1 (0.65%)	20 (11.24%)	76 (6.98%)	308 (10.8%)
Peripheral neuropathy ****	68 (9.87%)	91 (9.15%)	6 (1.43%)	-	-	17 (1.56%)	182 (6.4%)
Other types of pain *****	48 (6.97%)	54 (5.43%)	29 (6.90%)	10 (6.49%)	49 (27.53%)	25 (2.30%)	215 (7.5%)

* Includes SNX-5422, AT-13387, BIIB021, PU-H71, XL888, HSP990, KW-2478, Debio0932, TAS-116, LAQ824, Vorinostat + Paclitaxel and Bevacizumab. ** Includes myalgia and muscle pain. *** Includes abdominal pain, lower abdominal pain, upper abdominal pain, epigastric pain, gastrointestinal pain, and hepatic pain. **** Includes peripheral neuropathy, neuropathy, peripheral sensory neuropathy, and peripheral motor neuropathy. ***** Includes buttock pain, chest wall pain, hand foot syndrome, myalgia/arthralgia, oral pain, scalp pain, bladder pain, chest pain, eye pain, flank pain, musculoskeletal pain, non-cardiac chest pain, perineal pain, proctalgia, shoulder pain, stomach pain, tumor pain, infusion site pain, limb pain, neck pain, pelvic pain, lymph node pain, ear pain, urinary tract pain, joint pain, musculoskeletal motor neuropathy, toothache, and rectal pain. Hsp90: Heat shock protein 90; 17-AAG: 17-(Allylamino)-17-demethoxygeldanamycin; AUY922: luminespib; 17-DMAG: 17-dimethylaminoethylamino- 17-demethoxygeldanamycin. #: no data reported.

**Table 2:** Characteristics of randomized controlled trials.

Study ID	Date of Results Posted	Sample Size	Treatment	Control	Regimen	Indication
Concin et al. (2019) [23]	Aug. 2019	133	Ganetespib + Paclitaxel	Paclitaxel	Ganetespib: 150 mg/m^2^; Paclitaxel: 80 mg/m^2^	Epithelial ovarian cancer, fallopian tube cancer, and peritoneal cancer
Lee et al. (2016) [24]	Dec. 2016	133	Onalespib + crizotinib	Crizotinib	Onalespib: 220 mg/m^2^ i.v.; crizotinib: 250 mg oral	NSCLC
Lin et al. (2012) [25]	Jan. 2019	50	Ganetespib + fulvestrant	Fulvestrant	Ganetespib: 200 mg i.v.; Fulvestrant: 500 mg i.v.	Breast cancer
Synta Pharmaceuticals Corp. (2016) [26]	Jul. 2016	680	Ganetespib + docetaxel	Docetaxel	Ganetespib: 75 mg/m^2^; Docetaxel: 150 mg/m^2^	NSCLC
Ramalingam et al. (2015) [10]	Nov. 2015	249	Ganetespib + docetaxel	Docetaxel	Ganetespib: 150 mg/m^2^; docetaxel: 75 mg/m^2^	NSCLC

NSCLC: Non-small cell lung cancer.

## Data Availability

The data presented in this study are available in the article or Supplementary Table S1.

## Supplementary Materials

The following are available online at https://www.mdpi.com/1010-660X/57/1/5/s1, Table S1: Hsp90 inhibitor clinical trials with pain as adverse effects.
