# Supplementary material for: The Effect of Heat Shock Protein 90 Inhibitor on Pain in Cancer Patients: A Systematic Review and Meta-Analysis
Source: Medicina (Kaunas). 2020 Dec 23;57(1):5. doi: 10.3390/medicina57010005 (PMC7822414; doi:10.3390/medicina57010005)
Supplement: Supplementary file 1 [file medicina-57-00005-s001.pdf]

Supplemental Table S1. Clinical trials testing Heat shock protein 90 inhibitors

	Intervention	Phase	Randomization	Indication	Regimen	N	Treatment	Control	Pain event	# of Event
Ramalingam S et al. (2015) [1]	ganetespib	2	Yes	Advanced lung cancer and adenocarcinoma	150 mg/m^2, days 1 and 15, every 3 weeks	249	Docetaxel + Ganetespid (n = 123)	Docetaxel(n = 126)	Pain	Treatment: 24<br>Control: 12
Ronnen E et al. (2006) [2]	17-(Allylamino)-17-demethoxygeldanamycin (17-AGG)	2	No	papillary and clear cell renal cell carcinoma	220 mg/m^2 twice weekly for 2 weeks followed by a week of rest	20	17-AAG (n = 20)	No	Muscle weakness or pain	3
Pacey et al. (2011) [3]	17-dimethylaminoethylamino-17-demethoxygeldanamycin	1	No	Patients with advanced solid cancers	range 2.5 to 106 mg/m^2	25	17-DMAG (n = 25)	No	Joint pain	7
									Limb pain	1
									Headache	1
									Myalgia	2
									Abdomen pain	1
Pedersen KS et al. (2015) [4]	17-AAG and gemcitabine	2	No	Metastatic pancreatic adenocarcinoma	154 mg/m^2 on days 2 and 9 in 21 day cycles	20	17-AAG + gemcitabine (n = 20)	No	Abdominal pain	1
Lancet JE et al. (2010) [5]	17-dimethylaminoethylamino-17-demethoxygeldanamycin (17-DMAG or alvespimycin)	1	No	Acute myeloid leukemia (n = 23)<br>Chronic myelogenous leukemia-blast phase (n = 1)	8 mg/m^2 on days 1, 4, 8 and 11 every 3 weeks (doubled to 16 mg/m^2, then 32 mg/m^2, then reduced to 24 mg/m^2)	24	17-DMAG (n = 24)	No	Arthralgia	8mg/m2: 3<br>16mg/m2: 1<br>24mg/m2: 2<br>32mg/m2: 0
									Chest pain	8mg/m2: 2<br>16mg/m2: 2<br>24mg/m2: 0<br>32mg/m2: 0
Felip E et al. (2017) [6]	AUY922	2	No	advanced non-small cell lung cancer	70 mg/m^2 weekly	153	AUY922 (n = 153)	No	Back pain	31
									Abdominal pain	21
									Headache	52
									Myalgia	20
Slovin S et al. (2019) [7]	onalespib	1/2	Yes	adenocarcinoma of the prostate	220 mg/m^2 once weekly or 130 mg/m^2 twice weekly	48	onalespib weekly (n = 23)<br>onalespib twice weekly (n = 25)	No	Infusion site pain	7
									headache	6
Tse AN et al. (2008) [8]	irinotecan + 17-AAG	1	No	solid tumors refractory to standard treatment	220 mg/m2 17-AAG weekly titrated up to 450 mg/m^2 weekly	27	irinotecan + 17-AAG (n = 27)	No	Abdominal pain	3
									Neuropathy	1
Piotrowska Z et al. (2018) [9]	luminespib	2	No	epidermal growth factor receptor exon 20 insertions-positive non-small-cell lung cancer	70 mg/m^2 weekly	29	luminespib (n = 29)	No	Abdominal pain	4
									headache	4
Maddocks K et al. (2016) [10]	alvespimycin (17-DMAG)	1	No	chronic lymphocytic leukemia	16, 20 or 24 mg/m^2 days 1, 4, 8, 11 of 21 day cycle	15	17-DMAG	No	pain	3
Pacey S et al. (2012) [11]	17-AAG	2	No	metastatic malignant melanoma	450 mg/m^2	14	17-AAG	No	Back pain	1
									Chest pain	1
									Headache	6
									Muscle pain	3
Johnson M et al. (2015) [12]	AUY922 + erlotinib	1/2	No	adenocarcinoma of the lung NSCLC	25, 37.5, 55 or 70mg/m^2 weekly	37	AUY922 + Erlotinib (n = 38)	No	Myalgia/arthralgia	14

Bendell JC et al. (2015) [13]	AUY922 + capecitabine		1	No	metastatic or unresectable solid tumor	22, 28, 40, 55 or 70 mg/m^2	23	AUY922 + capecitabine (n = 23)	No	Hand foot syndrome	9
Jackman D et al. (2017) [14]	STA-9090 (ganetespib)		2	No	Relapsed or refractory small cell lung cancer	200 mg/m^2 weekly for 3 weeks, then week 4 off	25	STA-9090 (n = 25)	No	Abdominal pain	3
										Stomach pain	2
										Peripheral sensory neuropathy	2
Heath E et al. (2015) [15]	STA-9090 (ganetespib)		2	No	metastatic hormone-resistant prostate cancer	does not specify; once weekly for 3 weeks, off 4th week	18	STA-9090 (n = 18)	No	Abdominal pain	2
										Non-cardiac chest pain	3
										Pain	3
										Pain in extremity	4
										Bone pain	2
										Headache	2
										Peripheral sensory neuropathy	2
Oki Y et al. (2017) [16]	AUY922		2	No	lymphoma	70 mg/m^2 on Days 1, 8, 15 and 22 of 28 day cycle	20	AUY922 (n = 20)	No	Abdominal pain	5
										Oral pain	1
										Pain	2
										Back pain	1
										Bone pain	2
										Buttock pain	3
										Chest wall pain	1
										Myalgia	1
										Pain in extremity	7
										Peripheral sensory neuropathy	5
										Scalp pain	1
										Headache	4
Ray-Coquard I et al. (2019) [17]	STA-9090 (Ganetespib)		1	No	ovarian cancer	100 and 150 mg/m^2 weekly for 3 weeks, then 4th week off	10	ganetespib (n = 10)	No	Headache	3
										Peripheral neuropathy	2
										Abdominal pain	1
										Pain	0
										Polyneuropathy	0
Chen A et al. (2017) [18]		1	1	No	refractory solid tumors non-Hodgkin's Lymphoma	day 1 and 8 of 21 day cycle 10, 20, 40, 60, 80, 110, 150, 200, 266, 354, 470 mg/m^2	17	PU-H71 (n = 17)	No	Eye pain	1
										Abdominal pain	4
										Oral pain	1
										Rectal pain	1
										Stomach pain	1
										Toothace	1
										Flank pain	2
										Myalgia	2
										Pain in extremity	1
										Tumor pain	1
										Headache	3
										Pelvic pain	1
Isambert et al. (2015) [19]	Debio0932 (oral)		1	No	Advanced cancer	50 mg orally up to 1600 mg	50	Debio0932 (oral)	No	Upper abdominal pain	5
Do K et al. (2015) [20]	onalespib (AT13387)		1	No	Advanced Solid Tumors	20 mg/m^2 2 consecutive days each week for 3 weeks of a 4-week cycle (days 1, 2, 8, 9, 15 and 16). Max titration to 210 mg/m^2.	31	Onalespib (AT13387) is a second-generation, non-ansamycin	No	Abdominal pain	4

									Oral mucositis	2
Eroglu et al. (2018) [21]	Vemurafenib plus HSP90 inhibitor XL888	1	No	Unresectable or metastatic melanoma	Vemurafenib (960 mg po b.i.d.) plus XL888 (30, 45, 90, or 135 mg po twice weekly)	21	Vemurafenib plus HSP90 inhibitor XL888	No	Arthlagia	2
									Headache	2
									Pacreatitis	1
Oki et al. (2015) [22]	AUY922	1/2	No	Relapsed or refractory non-Hodgkin lymphoma	70 mg/m^2 over 2 hours on days 1, 8, 15 and 22 of 28-day cycles, for up to 12 cycles	20	AUY922	No	Abdominal pain	3
									Pain (extremity)	3
									Headache	2
									Non-neutopenic fever	3
Bendell et al. (2016) [23]	AUY922	2	No	Refractory Gastrointestinal Stromal Tumors	70 mg/m^2 by IV infusion over 60 minutes on days 1, 8 and 15 of each 21-day cycle.	25	AUY922	No	Abdominal pain	6
									Headache	6
									Pain	6
Cavenagh et al. (2017) [24]	KW-2478 (HSP90 inhibitor) plus bortezomib	1/2	No	Relapsed/refractory multiple myeloma	KW-2478 175 mg/m^2 plus BTZ 1.3mg/m^2 on days 1, 4, 8 and 11 every 3 weeks	95	KW-2478 (Hsp90 inhibitor) plus bortezomib	No	Peripheral Neuropathy	17
									Headache	10
Yong et al. (2015) [25]	KW-2478	1	No	Multiple myeloma and non-Hodgkin lymphoma	14 to176 mg/m^2 IV over 1 hour once daily on days 1–5	27	KW-2478	No	Headache	11
									Back pain	5
									Musculoskeletal chest pain	4
									Abdominal pain	3
Thakur et al. (2016) [26]	ganetespib	2	No	Patients with mCRPC who had been previously treated with docetaxel	200 mg/m^2 on days 1, 8 and 15 of every 28 days (one cycle)	17	ganetespib	No	Abdominal pain	2
Dickson et al. (2012) [27]	Oral BIIB021	2	No	Gastrointestinal Stromal Tumors	12 patients received 600 mg twice a week (BIW) and 11 patients received 400 mg three times a week	23	Oral BIIB021	No	Abdominal pain	1
Cercek et al. (2014) [28]	Ganetespib	2	No	Chemotherapy-refractory, metastatic colorectal cancer	200 mg/m^2 1 hour IV infusion	17	Ganetespib	No	Headache	4
Shapiro et al. (2014) [29]	AT13387	1	No	Advanced solid tumors	10 to 120 mg/m^2 twice weekly and 150 to 310 mg/m^2 once weekly (both for 3 weeks every 28 days).	62	AT13387	No	Abdominal pain	9
									Headache	9
Doi et al. (2014) [30]	AUY922	1	No	Japanese patients with advanced solid tumors	8 mg/m^2 starting dose IV once weekly for 28 day cycle	31	AUY922	No	Headache	3
Kong et al. (2016) [31]	AUY922 (HSP90 inhibitor) plus trastuzumab	1B/2	No	Metastatic HER2-positive breast cancer patients who have progressed on trastuzumab-based regimen	AUY922 dose at 55 mg/m^2 and 70 mg/m^2	45	AUY922 (HSP90 inhibitor) plus trastuzumab	No	Headache	7
									Abdominal pain	6
Wagner et al. (2013) [32]	Retaspimycin hydrochloride (IPI-504)	1	No	Gastrointestinal stromal tumors or soft tissue sarcomas	90 to 500 mg/m^2 IV twice weekly for 2 weeks on/1 week off	54	Retaspimycin hydrochloride (IPI-504)	No	Headache	27
									Arthlagia	16
									Myalgia	15

									Infusion site pain	14
									Back pain	12
Goldman et al. (2013) [33]	ganetespib (STA-9090)	1	No	Solid malignancies	7 to 259 mg/m^2 1 hour IV infusion once weekly for 3 weeks	53	ganetespib (STA-9090)	No	Abdominal pain	20
									Headache	9
									Back pain	8
									Musculoskeletal chest pain	6
									Extremity pain	6
Cardin et al. (2018) [34]	Ganetespib	2	No	Metastatic pancreatic cancer	175 mg/m^2 ganetespib IV once weekly for three weeks in four week cycles	14	Ganetespib	No	Abdominal pain	4
Sequist et al. (2010) [35]	IPI-504	2	No	Molecularly Defined Non–Small-Cell Lung Cancer	Starting dose was 400 mg/m^2 IV for 30 minutes on days 1, 4, 8 and 11 of a 21-day cycle, some evenutally lowered to 225 mg/m^2	76	IPI-504	No	Arthlagia	19
									Myalgia	19
									Headache	19
									Abdominal pain	18
									Back pain	16
									Infusion site pain	15
									Musculoskeletal chest pain	13
Saif et al. (2013) [36]	BIIB021	1	No	Advanced Solid Tumors	25 to 800 mg BIIB021 twice weekly	60	BIIB021	No	Headache	>12
Seggewiss-Bernhardt et al. (2015) [37]	NVP-AUY922 monotherapy or with Bortezomib	1/1b	No	Relapsed or Refractory Multiple Myeloma	8 to 70 mg/m^2	29	NVP-AUY922 monotherapy or with Bortezomib	No	Abdominal pain	3
Spreafico et al. (2015) [38]	HSP990 oral	1	No	Advanced solid malignancies	53 patients received HSP990 once weekly at 2.5, 5, 10, 20, 30, 50 or 60 mg and 11 patients received two times weekly at 25 mg.	64	HSP990 oral	No	Abdominal pain	10
									Headache	4
Rajan et al. (2011) [39]	PF-04929113 (SNX-5422)	1	No	Refractory solid tumor malignancies and lymphomas	Starting dose of 4 mg/m^2 orally twice weekly up to 177 mg/m^2 orally twice a week	33	PF-04929113 (SNX-5422)	No	Abdominal pain	1
									Joint pain	2
									Arthritis	2
									Muscle pain	1
Sequist L et al. (2013) [40]	IPI-504	2	No	lung cancer	225 mg/m^2 twice a week for 2 weeks followed by 10 days off therapy	3	IPI-504 (n = 3)	No	Arthralgia	1
Wagner A et al. (2016) [41]	onalespib (AT-13387) + imatinib	2	No	gastrointestinal stromal tumor	150, 180, 220 mg/m^2 weekly for 3 weeks 4th week off	26	onalespib + imatinib (n = 26)	No	Headache	5
Reddy N et al. (2013) [42]	SNX-5422 (PF-04929113)	1	No	hematological malignancy	5.32, 7, 9, 56, 74 mg/m^2 every other day for 21 days then 7 days off	25	PF-04929113 (n = 25)	No	Myalgia	1
Kummar et al. (2009) [43]	17-DMAG	1	No	Advanced malignancy	1, 2, 4, 6, 9, 12, 16, 21, 27 mg/m^2	31	17-DMAG	No	Musculoskeletal, motor neuropath	1
Shaw A et al. (2018) [44]	AUY922	2	No	Non-small cell lung cancer	not specified, infusion on Days 1, 8 and 15 of each 21 day cycle	6	AUY922 (n = 6)	No	Abdominal pain	3
									Myalgia	1

Hodi FS et al. (2018) [45]	STA-9090 (ganetespib)	2	No	ocular melanoma	200 mg/m^2 on days 1, 8, 15 of a 28 day cycle<br>150 mg/m^2 on days 1, 4, 8, 11, 15, 18 of a 28 day cycle	17	STA-9090 (n = 17)	No	Abdominal pain	2
									Back pain	6
									Eye pain	1
									Oral pain	1
									Non cardiac chest pain	2
									Pain	6
									Arthralgia	1
									Back pain	5
									chest wall pain	1
									Flank pain	1
									Pain in extremity	3
									Tumor pain	1
									Headache	8
									Peripheral sensory neuropathy	1
									Perineal pain	1
Lin N et al. (2019) [46]	fulvestrant +/- ganetespib	2	Yes	breast cancer	200 mg/m^2 on days 1, 8 and 15 of each 28 day cycle	50	fulvestrant + ganetespib (n = 35)<br>crossover from control to fulvestrant + ganetespib (n = 7)	fulvestrant 500 mg IM weekly (n = 15)	Hepatic pain	treatment: 1<br>control: 0<br>crossover: 0
									Peripheral motor neuropathy	treatment: 1<br>control: 0<br>crossover: 0
									Pain	treatment: 8<br>control: 4<br>crossover: 2
									Non cardiac chest pain	treatment: 3<br>control: 2<br>crossover: 0
									back pain	treatment: 11<br>control: 2<br>crossover: 0
									pain in extremity	treatment: 8<br>control: 5<br>crossover: 0
									arthralgia	treatment: 4<br>control: 4<br>crossover: 0
									bone pain	treatment: 2<br>control: 6<br>crossover: 0
									neck pain	treatment: 2<br>control: 3<br>crossover: 0
									Headache	treatment: 10<br>control: 3<br>crossover: 0
									Peripheral sensory neuropathy	treatment: 4<br>control: 2<br>crossover: 0

Meehan et al. (2018) [47]	Ganetespib + Ziv-Aflibercept	1	No	Refractory Gastrointestinal Carcinomas, Non-Squamous Non-Small Cell Lung Carcinomas, Urothelial Carcinomas, and Sarcomas	100 mg/m^2 weekly for 3 weeks, off 4th week	5	ganetespib + ziv aflibercept	No	abdominal pain	4
									gastrointestinal pain	1
									oral pain	1
									back pain	1
									shoulder pain	1
Modi S et al. (2013) [48]	retaspimycin (IPI-504) +trastuzumab	2	no	locally advanced or metastatic breast cancer	300 mg/m^2 once or twice weekly for 2 weeks, 3rd week off	26	retaspimycin + trastuzumab (*n* = 26)	no	headace	4
									abdominal pain	2
Infante J et al. (2014) [49]	SNX-5422	1	no	solid tumour or lymphoma not responsive to standard therapies	4-100 mg/m^2 QOD 21/28 days, 50-89 mg/m^2 QD 21/28 days, 50 mg/m^2 QD 28/28 days, 100-133 mg/m^2 QOD	56	SNX-5422	no	abdominal pain	8
Richardson PG et al. (2010) [50]	tanespimycin	1	no	relapsed multiple myeloma	150, 220, 275, 340, 420 and 525 mg/m^2 days 1, 4, 8 and 11 in each 3-week cycle	29	tanespimycin	no	back pain	10
									pain in extremity	6
									neuropathy	1
Burris HA et al. (2010) [51]	tanespimycin	1	yes	solid tumors	275 and 340 mg/m^2	17	tanespimycin (*n* = 17)	no	headache	6
Goyal L et al. (2015) [52]	ganetespib (STA-9090)	1	no	hepatocellular carcinoma	100 , 150 and 200 mg/m^2 IV given on days 1, 8 and 15 of each 28-day cycle.	14	ganetespib	no	headache	3
									abdominal pain	3
Saif et al (2013) [53]	BIIB021	1	no	advanced solid tumor	Schedule 1: 25 mg starting dose on day 1, 4, 8, 11, 15 and 18 of 28 day cycle + dose escalation<br>Schedule 2: 600 mg starting dose on day 1, 4, 8, 11, 15, 22 and 25 of 28 day cycle + dose escalation	60	BIIB021	no		
Modi et al (2011) [54]	trastuzamab and 17-AAG	2	no	HER2 positive breast cancer	450 mg/m^2 i.v. over 2 hours weekly	31	17-AAG	no	headache	16
									neuropathy	15
									arthralgia	7
									myalgia	7
Jhaveri K et al (2012) [55]	Trastuzumab and Alvespimycin	1	no	advanced solid tumors	60, 80 or 100 mg/m^2 IV weekly	28	alvespimycin	no	headache	14
									arthralgia	13
									myalgia	10
									back pain	7
Richardson PG et al. (2011) [56]	tanespimycin + bortezomib	1/2	No	refractory multiple myeloma	100-340 mg/m^2 days 1, 4, 8 and 11 in 21-day cycle.	72	tanespimycin + bortezomib (*n* = 72)	no	back pain	24
									peripheral neurpathy	21
Socinski MA et al. (2013) [57]	ganetespib	2	no	NSCLC	200 mg/m^2 weekly for 3 weeks then 1 week off	99	ganetespib	no	back pain	20
									headache	13
									abdominal pain	12
									arthralgia	10

Concin et al 2019 [58]	Paclitaxel +/- Ganestespib	1/2	Yes	Metastatic, p53-mutant, Platinum-resistant Ovarian Cancer	150 mg/m^2 weekly for 3 weeks off 1 week	133 ganetespib + paclitaxel (*n* = 90)	paclitaxel (*n* = 43)	abdominal pain	treatment: 34<br>control: 13
								arthralgia	treatment: 0<br>control: 2
								back pain	treatment: 9<br>control: 4
								myalgia	treatment: 0<br>control: 3
								pain in extremity	treatment: 9<br>control: 3
								neuropathy peripheral	treatment: 35<br>control: 21
Pillai RN et al. (2020) [59]	ganetespib +/- docetaxel	3	yes	advanced NSCLC	150 mg/m^2 days 1 and 15 of 21 day cycle	677 ganetespib + docetaxel (*n* = 335)	docetaxel (*n* = 337)	abdominal pain	treatment: 1<br>control: 0
								pain	treatment: 0<br>control: 1
								non cardiac chest pain	treatment: 0<br>control: 1
								musculoskeletal chest pain	treatment: 1<br>control: 0
								cancer pain	treatment: 1<br>control: 0
								Chest pain	treatment: 19<br>control: 20
								arthralgia	treatment: 17<br>control: 21
								back pain	treatment: 25<br>control: 15
								bone pain	treatment: 20<br>control: 17
								myalgia	treatment: 38<br>control: 28
								pain in extremity	treatment: 18<br>control: 16
								headache	treatment: 22<br>control: 17
								neuropathy peripheral	treatment: 41<br>control: 33
Lee JS et al. (2016) [60]	crizotinib +/- onalespib	2	yes	NSCLC	220 mg/m^2 weekly for 3 weeks then 1 week off	133 onalespib + crizotinib (*n* = 67)	crizotinib (*n* = 66)	headace	treatment: 18<br>control: 17
								abdominal pain upper	treatment: 11<br>control: 8
								abdominal pain	treatment: 14<br>control: 4

									myalgia	treatment: 12<br>control: 3
Gartner et al. (2012) [61]	17-allylamino-17-demethoxygeldanamycin	2	No	Metastatic or locally advanced, unresectable breast cancer	220 mg/m^2 IV over 2 hours on days 1, 4, 8 and 11 of a 21-day cycle.	11	17-allylamino-17-demethoxygeldanamycin	No	Chest pain	1
Kaufmann et al. (2011) [62]	cytarabine and tanespimycin	1	No	relapsed and refractory acute leukemia	cytarabine 400 mg/m^2/day continuously for 5 days and tanespimycin infusions at escalating doses on days 3 and 6.	26	cytarabine and tanespimycin	No	Myocardial infarction	1
Rampal et al. (2016) [63]	AUY922	2	No	myelofibrosis	55 and 70 mg/m^2 weekly	7	AUY922	no	pain	1
									back pain	1
									bone pain	1
									abdominal pain	2
									myalgia	1
									arthralgia	1
									peripheral sensory neuropathy	1
Speranze et al. (2018) [64]	PU-H71	1	No	Refractory Solid Tumors	IV over one hour on days 1 and 8 of 21 day cycles for varying amount of cycles (10 to 470 mg/m^2/day)	17	PU-H71	No	headache	1
Hong et al. (2013) [65]	BIIB028	1	No	Advanced Solid Tumors	(6- 192 mg/m^2) IV twice a week in 21 day cycles	41	BIIB028	No	headache	5
de Bono et al. (2008) [66]	LAQ824	1	No	Advanced Solid Tumors	(6-100 mg/m^2) IV 3-hour on days 1,2,3 for a 21 day cycle	39	LAQ824	No	headache	12
Ramaswamy et al. (2012) [67]	Vorinostat + Paclitaxel and Bevacizumab	1/2	No	Metastatic Breast Cancer	Vorinostat(200 or 300 mg PO BID) on days 1-3,8-10,15-17; paclitaxel (90 mg/m^2) days 2,9,16; Bevacizumab (10 mg/kg) days 2,16; 28 day cycle	54	Vorinostat + Paclitaxel and Bevacizumab	No	pain	39
									headache	22
									neuropathy	37
Ramanathan et al. (2010) [68]	17-DMAG	1	No	Advanced Solid tumors	Schedule A (16 mg/m^2 for 5 days) B (25 mg/m^2 for 3 days) 17-DMAG one hour IV for 3 weeks	56	17DMAG	No	none reported	
Oh et al. (2011) [69]	IPI-504	2	No	Castration-Resistant Prostate Cancer	IPI-504 IV (400 mg/m^2) on days 1,4,5,11 of 21 day cycle	19	IPI-504	No	arthralgia	5
									infusion site pain	4
									pain in extremity	4
									Musculoskeletal pain	3
									myalgia	3
									back pain	2
									Headache	2
Ramanathan et al. (2005) [70]	17-AAG	1	No	Refractory Advanced Cancers	(10-395 mg/m^2) IV 1 hour 3 times a week every 4 weeks	45	17-AAG	No	abdominal pain	2
									pancreatitus	2
Lyer et al. (2012) [71]	17-AAG and Docetaxel	1	No	Solid tumors	(55,70,57 mg/m^2) Docetaxel IV over 1 hour; (80-650 mg/m^2) 17-AAG IV 1-2 hour	49	Docetaxel and 17-AAG	No	abdominal pain	13
									chest pain	2
									myalgia	18
									arthralgia	11

									bone pain	7
									neuropathy	22
									headache	11
									tumor pain	16
Modi et al. (2007) [72]	17-AAG, KOS-953	1	No	Overexpressing Breast Cancer	(225,300,375,450 mg/m^2) Tanespimycin weekly; (2,4 mg/kg) Trastuzumab	25	Tanespimycin+Traztuzumab	No	headache	11
									abdominal pain	1
Heath E et al. (2017) [73]	17-AAG	2	No	Adenocarcinoma of the Prostate	300 mg/m^2 on days 1, 8 and 15 of 28 day cycle	17	17-AAG (tanespimycin)	no	abdominal pain	3
									back pain	4
									bone pain	2
									myalgia	1
									tumor pain	1
									headache	2
									peripheral motor neuropathy	1
									peripheral sensory neuropathy	1
Younes A et al. (2014) [74]	17-AAG	2	No	lymphoma	220 mg/m^2 days 1, 4, 8 and 11 of 21 day cycle	22	17-AAG (tanespimycin)	no	pain	5
									neuropathy	5
Kim et al. (2020) [75]	Sirolimus in combination with Ganetespib (SARC023)	1/2	No	Refractory Sarcomas and Malignant Peripheral Nerve Sheath Tumors	Ganetespib was administered intravenously (IV) over one hour on days 1, 8 and 15 of each 28-day cycle, and sirolimus administered orally once daily continuously after day 1 loading dose.	20	Sirolimus in combination with Ganetespib (SARC023)	no	Abdominal Pain	1
									Back pain	1
Jhaveri et al. (2014) [76]	Ganetespib (STA-9090)	2	No	Metastatic Breast Cancer	IV infusion of ganetespib weekly at a dose of 200 mg/m^2 over 1 hour for 3 consecutive weeks of a 28-day cycle.	22	Ganetespib (STA-9090)	No	Abdominal pain	1
Hendrickson et al. (2012) [77]	Gemcitabine in Combination with Tanespimycin	2	No	Advanced Epithelial Ovarian and Primary Peritoneal Carcinoma	tanespimycin 154 mg/m^2 on days 1 and 9 of cycle 1 and days 2 and 9 of subsequent cycles. Patients also received gemcitabine 750 mg/m^2 on day 8 of the first treatment cycle and days 1 and 8 of subsequent cycles.	29	Gemcitabine in Combination with Tanespimycin	No	Headache	1
									Abdominal Pain	3
									Stomach pain	1
Linehan WM et al. (2011) [78]	17-AAG	2	No	Hippel Lindau disease and kidney cancer	300 mg/m^2 days 1, 8 and 15 of 28 cycles	9	17-AAG	No	abdominal pain	2
									back pain	1
									bone pain	1
									chest wall pain	1
									limb pain	1
									muscle pain	3
									neck pain	1
									pelvis pain	1
									headache	5
									pain	1

Subramaniam et al. (2018) [79]	ganetespib + doxorubicin	1/2	No	small cell lung cancer	100 or 150 mg/m^2 on days 1 and 8 of 21 day cycle	11	ganetespib + doxorubicin	No	epigastric pain	1
									headache	1
									pain	2
Synta Pharmaceuticals Corp. (2016) [80]	STA-9090	2	No	gastrointestinal stromal tumor	200 mg/m^2 weekly for 3 weeks off 1 week	27	ganetespib	No	serious abdominal pain	2
									hepatic pain	1
									eye pain	1
									abdominal pain	5
									lower abdominal pain	4
									upper abdominal pain	2
									proctalgia	1
									chest pain	1
									arthralgia	6
									back pain	6
									myalgia	5
									pain in extremity	2
									flank pain	1
									Musculoskeletal pain	1
									tumor pain	1
									headache	10
									peripheral sensory neuropathy	2
									peripheral motor neuropathy	1
									bladder pain	1
Banerji et al. (2005) [81]	17-Allylamino, 17-Demethoxygeldanamycin	1	No	Advanced Malignancies	10 to 450 mg/m^2/week	30	17-Allylamino, 17-Demethoxygeldanamycin	No	Headache	1
Heath, et al. (2008) [82]	17-AAG	2	No	Hormone-Refractory Metastatic Prostate Cancer	300 mg/m^2 IV weekly for three out of four weeks	15	17-AAG	No	Back pain	2
									Abdominal pain	1
Solit et al. (2008) [83]	17-Allylamino-17-Demethoxygeldanamycin	2	No	Metastatic Melanoma	450 mg/m^2 IV once weekly for 6 weeks	15	17-Allylamino-17-Demethoxygeldanamycin	No	Musculoskeletal pain	2
									Fever	1
									Cardiac ischemia	1
Cercek et al. (2015) [84]	STA-9090 (ganetespib)	2	No	Refractory metastatic colorectal cancer	200 mg/m^2 weekly for 3 weeks, 1 week off	15	STA-9090 (ganetespib)	No	serious abdominal pain	2
									non-serious abdominal pain	4
Vaishampayan UN et al. (2011) [85]	Sorafenib and tanespimycin	1	No	solid tumor malignancy	300, 350, 400 or 450 mg/m^2 weekly for 3 weeks off 1 week	27	sorafenib and tanespimycin	no	headache	6
									myalgia	3
									abdominal pain	3
Ramalingam et al. (2008) [86]	17-AAG and paclitaxel	1	no	advanced solid malignancies	80, 100, 130 or 175 mg/m^2 twice weekly for 3 weeks, off 1 week	25	17-AAG and paclitaxel	no	abdominal pain	1
									arthralgia	1
									chest pain	1
									headache	1
									myalgia	2
									pain	1
									peripheral sensory neuropathy	1
Orlemans et al. (2015) [87]	SNX-5422	1/2	No	HER2 Positive Cancers	100 mg/m^2 capsules every other day for 21 days (total = 11 doses),	15	SNX-5422	No	Upper abdominal pain	2
									Abdominal pain	3
									Arthrlagia	1
									Back pain	1

									Headache	1
Moley et al. (2017) [88]	17-Allylaminogeldanamycin (17AAG)	2	No	Inoperable Locoregionally Advanced or Metastatic Thyroid Cancer	220 mg/m^2 tanespimycin IV over 2-6 hours on days 1, 4, 8 and 11. Courses repeat every 21 days in the absence of disease progression or unacceptable toxicity.	41	17-AAG	No	Abdominal pain	1
									Fever	4
									Headache	7
									Lymph node pain	2
									Ear pain	1
									Chest pain	1
									Pain	3
									Arthralgia	4
									Back pain	5
									Bone pain	5
									Myalgia	8
									Neck pain	5
									Pain in extremity	1
Gartner et al. (2017) [89]	17-AAG	2	no	Refractory Locally Advanced or Metastatic Breast Cancer	tanespimycin IV over 1-6 hours on days 1, 4, 8 and 11	11	17-AAG	no	Abdominal pain	1
									Pain in extremity	1
									Arthralgia	1
									Bone pain	1
									Myalgia	1
									Headache	2
									Urinary tract pain	1
Weigel et al (2007) [90]	17-AAG	1	no	Pediatric Patients with SolidTumors	150, 200, 270 and 360 mg/m^2 days 1, 4, 8 and 11of a 21-day cycle	12	17-AAG	no	abdominal pain	1
									pain in extremity	1
									headache	1
									pain	1

References

1. Ramalingam, S.; Goss, G.; Rosell, R.; Schmid-Bindert, G.; Zaric, B.; Andric, Z.; Bondarenko, I.; Komov, D.; Ceric, T.; Khuri, F., et al. A randomized phase II study of ganetespib, a heat shock protein 90 inhibitor, in combination with docetaxel in second-line therapy of advanced non-small cell lung cancer (GALAXY-1). *Ann Oncol* **2015**, *26*, 1741-1748, doi:10.1093/annonc/mdv220.
2. Ronnen, E.A.; Kondagunta, G.V.; Ishill, N.; Sweeney, S.M.; Deluca, J.K.; Schwartz, L.; Bacik, J.; Motzer, R.J. A phase II trial of 17-(Allylamino)-17-demethoxygeldanamycin in patients with papillary and clear cell renal cell carcinoma. *Investigational new drugs* **2006**, *24*, 543-546, doi:10.1007/s10637-006-9208-z.
3. Pacey, S.; Wilson, R.H.; Walton, M.; Eatock, M.M.; Hardcastle, A.; Zetterlund, A.; Arkenau, H.T.; Moreno-Farre, J.; Banerji, U.; Roels, B., et al. A phase I study of the heat shock protein 90 inhibitor alvespimycin (17-DMAG) given intravenously to patients with advanced solid tumors. *Clinical cancer research : an official journal of the American Association for Cancer Research* **2011**, *17*, 1561-1570, doi:10.1158/1078-0432.CCR-10-1927.
4. Pedersen, K.S.; Kim, G.P.; Foster, N.R.; Wang-Gillam, A.; Erlichman, C.; McWilliams, R.R. Phase II trial of gemcitabine and tanespimycin (17AAG) in metastatic pancreatic cancer: a Mayo Clinic Phase II Consortium study. *Investigational new drugs* **2015**, *33*, 963-968, doi:10.1007/s10637-015-0246-2.
5. Lancet, J.E.; Gojo, I.; Burton, M.; Quinn, M.; Tighe, S.M.; Kersey, K.; Zhong, Z.; Albitar, M.X.; Bhalla, K.; Hannah, A.L., et al. Phase I study of the heat shock protein 90 inhibitor alvespimycin (KOS-1022, 17-DMAG) administered intravenously twice weekly to patients with acute myeloid leukemia. *Leukemia* **2010**, *24*, 699-705, doi:10.1038/leu.2009.292.
6. Felip, E.; Barlesi, F.; Besse, B.; Chu, Q.; Gandhi, L.; Kim, S.W.; Carcereny, E.; Sequist, L.V.; Brunsvig, P.; Chouaid, C., et al. Phase 2 Study of the HSP-90 Inhibitor AUY922 in Previously Treated and Molecularly Defined Patients with Advanced Non-Small Cell Lung Cancer. *J Thorac Oncol* **2018**, *13*, 576-584, doi:10.1016/j.jtho.2017.11.131.
7. Slovin, S.; Hussain, S.; Saad, F.; Garcia, J.; Picus, J.; Ferraldeschi, R.; Crespo, M.; Flohr, P.; Riisnaes, R.; Lin, C., et al. Pharmacodynamic and Clinical Results from a Phase I/II Study of the HSP90 Inhibitor Onalespib in Combination with Abiraterone Acetate in Prostate Cancer. *Clinical cancer research : an official journal of the American Association for Cancer Research* **2019**, *25*, 4624-4633, doi:10.1158/1078-0432.CCR-18-3212.
8. Tse, A.N.; Klimstra, D.S.; Gonen, M.; Shah, M.; Sheikh, T.; Sikorski, R.; Carvajal, R.; Mui, J.; Tipian, C.; O'Reilly, E., et al. A phase 1 dose-escalation study of irinotecan in combination with 17-allylamino-17-demethoxygeldanamycin in patients with solid tumors. *Clinical cancer research : an official journal of the American Association for Cancer Research* **2008**, *14*, 6704-6711, doi:10.1158/1078-0432.CCR-08-1006.
9. Piotrowska, Z.; Costa, D.B.; Oxnard, G.R.; Huberman, M.; Gainor, J.F.; Lennes, I.T.; Muzikansky, A.; Shaw, A.T.; Azzoli, C.G.; Heist, R.S., et al. Activity of the Hsp90 inhibitor luminespib among non-small-cell lung cancers harboring EGFR exon 20 insertions. *Ann Oncol* **2018**, *29*, 2092-2097, doi:10.1093/annonc/mdy336.
10. Maddocks, K.; Hertlein, E.; Chen, T.L.; Wagner, A.J.; Ling, Y.; Flynn, J.; Phelps, M.; Johnson, A.J.; Byrd, J.C.; Jones, J.A. A phase I trial of the intravenous Hsp90 inhibitor alvespimycin (17-DMAG) in patients with relapsed chronic lymphocytic leukemia/small lymphocytic lymphoma. *Leukemia & lymphoma* **2016**, *57*, 2212-2215, doi:10.3109/10428194.2015.1129536.
11. Pacey, S.; Gore, M.; Chao, D.; Banerji, U.; Larkin, J.; Sarker, S.; Owen, K.; Asad, Y.; Raynaud, F.; Walton, M., et al. A Phase II trial of 17-allylamino, 17-demethoxygeldanamycin (17-AAG, tanespimycin) in patients with metastatic melanoma. *Investigational new drugs* **2012**, *30*, 341-349, doi:10.1007/s10637-010-9493-4.
12. Johnson, M.L.; Yu, H.A.; Hart, E.M.; Weitner, B.B.; Rademaker, A.W.; Patel, J.D.; Kris, M.G.; Riely, G.J. Phase I/II Study of HSP90 Inhibitor AUY922 and Erlotinib for EGFR-Mutant Lung Cancer With Acquired Resistance to Epidermal Growth Factor Receptor Tyrosine Kinase Inhibitors. *J Clin Oncol* **2015**, *33*, 1666-1673, doi:10.1200/JCO.2014.59.7328.

13. Bendell, J.C.; Jones, S.F.; Hart, L.; Pant, S.; Moyhuddin, A.; Lane, C.M.; Earwood, C.; Murphy, P.; Patton, J.; Penley, W.C., et al. A Phase I Study of the Hsp90 Inhibitor AUY922 plus Capecitabine for the Treatment of Patients with Advanced Solid Tumors. *Cancer Investigation* **2015**, *33*, 477-482, doi:10.3109/07357907.2015.1069834.
14. Jackman, D.M. Study of Hsp90 Inhibitor, STA-9090 for Relapsed or Refractory Small Cell Lung Cancer. **2017**, https://www.clinicaltrials.gov/ct2/show/NCT01173523?term=STA-01179090&cond=small+cell+lung+cancer&draw=01173522&rank=01173521.
15. Heath, E. Hsp90 Inhibitor STA-9090 in Treating Patients With Metastatic Hormone-Resistant Prostate Cancer Previously Treated With Docetaxel-Based Chemotherapy. **2015**, https://www.clinicaltrials.gov/ct2/show/NCT01270880?term=ganetespib+heath&draw=01270882&rank=01270881.
16. Oki, Y. A Study of the HSP90 Inhibitor AUY922. **2017**, https://www.clinicaltrials.gov/ct2/show/NCT01485536?term=AUY01485922&draw=01485532&rank=01485536.
17. Ray-Coquard, I.; Braicu, I.; Berger, R.; Mahner, S.; Sehouli, J.; Pujade-Lauraine, E.; Cassier, P.A.; Moll, U.M.; Ulmer, H.; Leunen, K., et al. Part I of GANNET53: A European Multicenter Phase I/II Trial of the Hsp90 Inhibitor Ganetespib Combined With Weekly Paclitaxel in Women With High-Grade, Platinum-Resistant Epithelial Ovarian Cancer-A Study of the GANNET53 Consortium. *Front Oncol* **2019**, *9*, 832, doi:10.3389/fonc.2019.00832.
18. Chen, A. PU-H71 in Patients With Solid Tumors and Low-Grade Non-Hodgkin's Lymphoma That Have Not Responded to Standard Treatment. **2017**, https://www.clinicaltrials.gov/ct2/show/NCT01581541?term=PU-H01581571&draw=01581542&rank=01581545.
19. Isambert, N.; Delord, J.P.; Soria, J.C.; Hollebecque, A.; Gomez-Roca, C.; Purcea, D.; Rouits, E.; Belli, R.; Fumoleau, P. Debio0932, a second-generation oral heat shock protein (HSP) inhibitor, in patients with advanced cancer-results of a first-in-man dose-escalation study with a fixed-dose extension phase. *Ann Oncol* **2015**, *26*, 1005-1011, doi:10.1093/annonc/mdv031.
20. Do, K.; Speranza, G.; Chang, L.C.; Polley, E.C.; Bishop, R.; Zhu, W.; Trepel, J.B.; Lee, S.; Lee, M.J.; Kinders, R.J., et al. Phase I study of the heat shock protein 90 (Hsp90) inhibitor onalespib (AT13387) administered on a daily for 2 consecutive days per week dosing schedule in patients with advanced solid tumors. *Investigational new drugs* **2015**, *33*, 921-930, doi:10.1007/s10637-015-0255-1.
21. Eroglu, Z.; Chen, Y.A.; Gibney, G.T.; Weber, J.S.; Kudchadkar, R.R.; Khushalani, N.I.; Markowitz, J.; Brohl, A.S.; Tetteh, L.F.; Ramadan, H., et al. Combined BRAF and HSP90 Inhibition in Patients with Unresectable BRAF (V600E)-Mutant Melanoma. *Clinical cancer research : an official journal of the American Association for Cancer Research* **2018**, *24*, 5516-5524, doi:10.1158/1078-0432.CCR-18-0565.
22. Oki, Y.; Younes, A.; Knickerbocker, J.; Samaniego, F.; Nastoupil, L.; Hagemeister, F.; Romaguera, J.; Fowler, N.; Kwak, L.; Westin, J. Experience with HSP90 inhibitor AUY922 in patients with relapsed or refractory non-Hodgkin lymphoma. *haematologica* **2015**, *100*, e272.
23. Bendell, J.C.; Bauer, T.M.; Lamar, R.; Joseph, M.; Penley, W.; Thompson, D.S.; Spigel, D.R.; Owera, R.; Lane, C.M.; Earwood, C., et al. A Phase 2 Study of the Hsp90 Inhibitor AUY922 as Treatment for Patients with Refractory Gastrointestinal Stromal Tumors. *Cancer Invest* **2016**, *34*, 265-270, doi:10.1080/07357907.2016.1193746.
24. Cavenagh, J.; Oakervee, H.; Baetiong-Caguioa, P.; Davies, F.; Gharibo, M.; Rabin, N.; Kurman, M.; Novak, B.; Shiraishi, N.; Nakashima, D., et al. A phase I/II study of KW-2478, an Hsp90 inhibitor, in combination with bortezomib in patients with relapsed/refractory multiple myeloma. *British journal of cancer* **2017**, *117*, 1295-1302, doi:10.1038/bjc.2017.302.

25. Yong, K.; Cavet, J.; Johnson, P.; Morgan, G.; Williams, C.; Nakashima, D.; Akinaga, S.; Oakervee, H.; Cavenagh, J. Phase I study of KW-2478, a novel Hsp90 inhibitor, in patients with B-cell malignancies. *British journal of cancer* **2016**, *114*, 7-13, doi:10.1038/bjc.2015.422.
26. Thakur, M.K.; Heilbrun, L.K.; Sheng, S.; Stein, M.; Liu, G.; Antonarakis, E.S.; Vaishampayan, U.; Dzinic, S.H.; Li, X.; Freeman, S., et al. A phase II trial of ganetespib, a heat shock protein 90 Hsp90) inhibitor, in patients with docetaxel-pretreated metastatic castrate-resistant prostate cancer (CRPC)-a prostate cancer clinical trials consortium (PCCTC) study. *Investigational new drugs* **2016**, *34*, 112-118, doi:10.1007/s10637-015-0307-6.
27. Dickson, M.A.; Okuno, S.H.; Keohan, M.L.; Maki, R.G.; D'Adamo, D.R.; Akhurst, T.J.; Antonescu, C.R.; Schwartz, G.K. Phase II study of the HSP90-inhibitor BIIB021 in gastrointestinal stromal tumors. *Ann Oncol* **2013**, *24*, 252-257, doi:10.1093/annonc/mds275.
28. Cercek, A.; Shia, J.; Gollub, M.; Chou, J.F.; Capanu, M.; Raasch, P.; Reidy-Lagunes, D.; Proia, D.A.; Vakiani, E.; Solit, D.B., et al. Ganetespib, a novel Hsp90 inhibitor in patients with KRAS mutated and wild type, refractory metastatic colorectal cancer. *Clin Colorectal Cancer* **2014**, *13*, 207-212, doi:10.1016/j.clcc.2014.09.001.
29. Shapiro, G.I.; Kwak, E.; Dezube, B.J.; Yule, M.; Ayrton, J.; Lyons, J.; Mahadevan, D. First-in-human phase I dose escalation study of a second-generation non-ansamycin HSP90 inhibitor, AT13387, in patients with advanced solid tumors. *Clinical cancer research : an official journal of the American Association for Cancer Research* **2015**, *21*, 87-97, doi:10.1158/1078-0432.CCR-14-0979.
30. Doi, T.; Onozawa, Y.; Fuse, N.; Yoshino, T.; Yamazaki, K.; Watanabe, J.; Akimov, M.; Robson, M.; Boku, N.; Ohtsu, A. Phase I dose-escalation study of the HSP90 inhibitor AUY922 in Japanese patients with advanced solid tumors. *Cancer chemotherapy and pharmacology* **2014**, *74*, 629-636.
31. Kong, A.; Rea, D.; Ahmed, S.; Beck, J.T.; López, R.L.; Biganzoli, L.; Armstrong, A.C.; Aglietta, M.; Alba, E.; Campone, M. Phase 1B/2 study of the HSP90 inhibitor AUY922 plus trastuzumab in metastatic HER2-positive breast cancer patients who have progressed on trastuzumab-based regimen. *Oncotarget* **2016**, *7*, 37680.
32. Wagner, A.J.; Chugh, R.; Rosen, L.S.; Morgan, J.A.; George, S.; Gordon, M.; Dunbar, J.; Normant, E.; Grayzel, D.; Demetri, G.D. A phase I study of the HSP90 inhibitor retaspimycin hydrochloride (IPI-504) in patients with gastrointestinal stromal tumors or soft-tissue sarcomas. *Clinical cancer research : an official journal of the American Association for Cancer Research* **2013**, *19*, 6020-6029, doi:10.1158/1078-0432.CCR-13-0953.
33. Goldman, J.W.; Raju, R.N.; Gordon, G.A.; El-Hariry, I.; Teofilivici, F.; Vukovic, V.M.; Bradley, R.; Karol, M.D.; Chen, Y.; Guo, W., et al. A first in human, safety, pharmacokinetics, and clinical activity phase I study of once weekly administration of the Hsp90 inhibitor ganetespib (STA-9090) in patients with solid malignancies. *BMC Cancer* **2013**, *13*, 152, doi:10.1186/1471-2407-13-152.
34. Cardin, D.B.; Thota, R.; Goff, L.W.; Berlin, J.D.; Jones, C.M.; Ayers, G.D.; Whisenant, J.G.; Chan, E. A Phase II Study of Ganetespib as Second-line or Third-line Therapy for Metastatic Pancreatic Cancer. *American journal of clinical oncology* **2018**, *41*, 772-776, doi:10.1097/coc.0000000000000377.
35. Sequist, L.V.; Gettinger, S.; Senzer, N.N.; Martins, R.G.; Janne, P.A.; Lilenbaum, R.; Gray, J.E.; Iafrate, A.J.; Katayama, R.; Hafeez, N., et al. Activity of IPI-504, a novel heat-shock protein 90 inhibitor, in patients with molecularly defined non-small-cell lung cancer. *J Clin Oncol* **2010**, *28*, 4953-4960, doi:10.1200/JCO.2010.30.8338.
36. Saif, M.W.; Erlichman, C.; Dragovich, T.; Mendelson, D.; Toft, D.; Burrows, F.; Storgard, C.; Von Hoff, D. Open-label, dose-escalation, safety, pharmacokinetic, and pharmacodynamic study of intravenously administered CNF1010 (17-(allylamino)-17-demethoxygeldanamycin [17-AAG]) in patients with solid tumors. *Cancer chemotherapy and pharmacology* **2013**, *71*, 1345-1355, doi:10.1007/s00280-013-2134-9.

37. Seggewiss-Bernhardt, R.; Bargou, R.C.; Goh, Y.T.; Stewart, A.K.; Spencer, A.; Alegre, A.; Blade, J.; Ottmann, O.G.; Fernandez-Ibarra, C.; Lu, H., et al. Phase 1/1B trial of the heat shock protein 90 inhibitor NVP-AUY922 as monotherapy or in combination with bortezomib in patients with relapsed or refractory multiple myeloma. *Cancer* **2015**, *121*, 2185-2192, doi:10.1002/cncr.29339.
38. Spreafico, A.; Delord, J.P.; De Mattos-Arruda, L.; Berge, Y.; Rodon, J.; Cottura, E.; Bedard, P.L.; Akimov, M.; Lu, H.; Pain, S., et al. A first-in-human phase I, dose-escalation, multicentre study of HSP990 administered orally in adult patients with advanced solid malignancies. *British journal of cancer* **2015**, *112*, 650-659, doi:10.1038/bjc.2014.653.
39. Rajan, A.; Kelly, R.J.; Trepel, J.B.; Kim, Y.S.; Alarcon, S.V.; Kummar, S.; Gutierrez, M.; Crandon, S.; Zein, W.M.; Jain, L., et al. A phase I study of PF-04929113 (SNX-5422), an orally bioavailable heat shock protein 90 inhibitor, in patients with refractory solid tumor malignancies and lymphomas. *Clinical cancer research : an official journal of the American Association for Cancer Research* **2011**, *17*, 6831-6839, doi:10.1158/1078-0432.CCR-11-0821.
40. Sequist, L.V. IPI-504 in NSCLC Patients With ALK Translocations. **2013**, https://www.clinicaltrials.gov/ct2/show/NCT01228435?term=IPI-01228504&cond=lung+cancer&draw=01228432&rank=01228434.
41. Wagner, A.J.; Agulnik, M.; Heinrich, M.C.; Mahadevan, D.; Riedel, R.F.; von Mehren, M.; Trent, J.; Demetri, G.D.; Corless, C.L.; Yule, M., et al. Dose-escalation study of a second-generation non-ansamycin HSP90 inhibitor, onalespib (AT13387), in combination with imatinib in patients with metastatic gastrointestinal stromal tumour. *European journal of cancer (Oxford, England : 1990)* **2016**, *61*, 94-101, doi:10.1016/j.ejca.2016.03.076.
42. Reddy, N.; Voorhees, P.M.; Houk, B.E.; Brega, N.; Hinson, J.M., Jr.; Jillela, A. Phase I trial of the HSP90 inhibitor PF-04929113 (SNX5422) in adult patients with recurrent, refractory hematologic malignancies. *Clin Lymphoma Myeloma Leuk* **2013**, *13*, 385-391, doi:10.1016/j.clml.2013.03.010.
43. Kummar, S.; Gutierrez, M.E.; Gardner, E.R.; Chen, X.; Figg, W.D.; Zajac-Kaye, M.; Chen, M.; Steinberg, S.M.; Muir, C.A.; Yancey, M.A., et al. Phase I trial of 17-dimethylaminoethylamino-17-demethoxygeldanamycin (17-DMAG), a heat shock protein inhibitor, administered twice weekly in patients with advanced malignancies. *European journal of cancer (Oxford, England : 1990)* **2010**, *46*, 340-347, doi:10.1016/j.ejca.2009.10.026.
44. Socinski, M.A.; Goldman, J.; El-Hariry, I.; Koczywas, M.; Vukovic, V.; Horn, L.; Paschold, E.; Salgia, R.; West, H.; Sequist, L.V., et al. A multicenter phase II study of ganetespib monotherapy in patients with genotypically defined advanced non-small cell lung cancer. *Clinical cancer research : an official journal of the American Association for Cancer Research* **2013**, *19*, 3068-3077, doi:10.1158/1078-0432.CCR-12-3381.
45. Hodi, F.S. STA-9090(Ganetespib) in Metastatic Ocular Melanoma. **2018**, https://www.clinicaltrials.gov/ct2/show/NCT01200238?term=STA-01209090&cond=ocular&draw=01200232&rank=01200231.
46. Lin, N.U. Fulvestrant With or Without Ganetespib in HR+ Breast Cancer. **2019**, https://www.clinicaltrials.gov/ct2/show/NCT01560416?term=ganetespib+fulvestrant&draw=01560412&rank=01560411.
47. Meehan, R.; Kummar, S.; Do, K.; O'Sullivan Coyne, G.; Juwara, L.; Zlott, J.; Rubinstein, L.; Doroshow, J.H.; Chen, A.P. A Phase I Study of Ganetespib and Ziv-Aflibercept in Patients with Advanced Carcinomas and Sarcomas. *Oncologist* **2018**, *23*, 1269-e1125, doi:10.1634/theoncologist.2018-0203.
48. Modi, S.; Saura, C.; Henderson, C.; Lin, N.U.; Mahtani, R.; Goddard, J.; Rodenas, E.; Hudis, C.; O'Shaughnessy, J.; Baselga, J. A multicenter trial evaluating retaspimycin HCL (IPI-504) plus trastuzumab in patients with advanced or metastatic HER2-positive breast cancer. *Breast Cancer Res Treat* **2013**, *139*, 107-113, doi:10.1007/s10549-013-2510-5.

49. Infante, J.R.; Weiss, G.J.; Jones, S.; Tibes, R.; Bauer, T.M.; Bendell, J.C.; Hinson, J.M., Jr.; Von Hoff, D.D.; Burris, H.A., 3rd; Orlemans, E.O., et al. Phase I dose-escalation studies of SNX-5422, an orally bioavailable heat shock protein 90 inhibitor, in patients with refractory solid tumours. *European journal of cancer (Oxford, England : 1990)* **2014**, *50*, 2897-2904, doi:10.1016/j.ejca.2014.07.017.
50. Richardson, P.G.; Chanan-Khan, A.A.; Alsina, M.; Albitar, M.; Berman, D.; Messina, M.; Mitsiades, C.S.; Anderson, K.C. Tanespimycin monotherapy in relapsed multiple myeloma: results of a phase 1 dose-escalation study. *British journal of haematology* **2010**, *150*, 438-445, doi:10.1111/j.1365-2141.2010.08265.x.
51. Burris, H.A., 3rd; Berman, D.; Murthy, B.; Jones, S. Tanespimycin pharmacokinetics: a randomized dose-escalation crossover phase 1 study of two formulations. *Cancer chemotherapy and pharmacology* **2011**, *67*, 1045-1054, doi:10.1007/s00280-010-1398-6.
52. Goyal, L.; Wadlow, R.C.; Blaszkowsky, L.S.; Wolpin, B.M.; Abrams, T.A.; McCleary, N.J.; Sheehan, S.; Sundaram, E.; Karol, M.D.; Chen, J., et al. A phase I and pharmacokinetic study of ganetespib (STA-9090) in advanced hepatocellular carcinoma. *Investigational new drugs* **2015**, *33*, 128-137, doi:10.1007/s10637-014-0164-8.
53. Saif, M.W.; Takimoto, C.; Mita, M.; Banerji, U.; Lamanna, N.; Castro, J.; O'Brien, S.; Stogard, C.; Von Hoff, D. A phase 1, dose-escalation, pharmacokinetic and pharmacodynamic study of BIIB021 administered orally in patients with advanced solid tumors. *Clinical cancer research : an official journal of the American Association for Cancer Research* **2014**, *20*, 445-455, doi:10.1158/1078-0432.CCR-13-1257.
54. Modi, S.; Stopeck, A.; Linden, H.; Solit, D.; Chandarlapaty, S.; Rosen, N.; D'Andrea, G.; Dickler, M.; Moynahan, M.E.; Sugarman, S., et al. HSP90 inhibition is effective in breast cancer: a phase II trial of tanespimycin (17-AAG) plus trastuzumab in patients with HER2-positive metastatic breast cancer progressing on trastuzumab. *Clinical cancer research : an official journal of the American Association for Cancer Research* **2011**, *17*, 5132-5139, doi:10.1158/1078-0432.ccr-11-0072.
55. Jhaveri, K.; Miller, K.; Rosen, L.; Schneider, B.; Chap, L.; Hannah, A.; Zhong, Z.; Ma, W.; Hudis, C.; Modi, S. A phase I dose-escalation trial of trastuzumab and alvespimycin hydrochloride (KOS-1022; 17 DMAG) in the treatment of advanced solid tumors. *Clinical cancer research : an official journal of the American Association for Cancer Research* **2012**, *18*, 5090-5098, doi:10.1158/1078-0432.ccr-11-3200.
56. Richardson, P.G.; Chanan-Khan, A.A.; Lonial, S.; Krishnan, A.Y.; Carroll, M.P.; Alsina, M.; Albitar, M.; Berman, D.; Messina, M.; Anderson, K.C. Tanespimycin and bortezomib combination treatment in patients with relapsed or relapsed and refractory multiple myeloma: results of a phase 1/2 study. *British journal of haematology* **2011**, *153*, 729-740, doi:10.1111/j.1365-2141.2011.08664.x.
57. Shaw, A. AUY922 for Advanced ALK-positive NSCLC. **2018**, https://www.clinicaltrials.gov/ct2/show/NCT01752400?term=AUY01752922&cond=Lung&draw=01752402&rank=01752405.
58. Concin, N. GANNET53: Ganetespib in Metastatic, p53-mutant, Platinum-resistant Ovarian Cancer. **2019**, https://www.clinicaltrials.gov/ct2/show/study/NCT02012192?term=concin&draw=02012192&rank=02012191.
59. Pillai, R.N.; Fennell, D.A.; Kovcin, V.; Ciuleanu, T.-E.; Ramlau, R.; Kowalski, D.; Schenker, M.; Yalcin, I.; Teofilovici, F.; Vukovic, V.M. Randomized Phase III Study of Ganetespib, a Heat Shock Protein 90 Inhibitor, With Docetaxel Versus Docetaxel in Advanced Non–Small-Cell Lung Cancer (GALAXY-2). *Journal of Clinical Oncology* **2020**, *38*, 613-622.
60. Lee, J.-S.; Han, J.-Y.; Ahn, M.-J.; Oh, I.-J.; Kim, H.; Lee, D.H.; Bertino, E.M.; Ramirez, S.V.; Pennell, N.A.; Wozniak10, A.J. Addition of HSP90 inhibitor onalespib to crizotinib prior to progression in patients with ALK-pos NSCLC; results of a randomized Phase 2 study. *Blood* **2016**, *3*, 3.8.
61. Gartner, E.M.; Silverman, P.; Simon, M.; Flaherty, L.; Abrams, J.; Ivy, P.; Lorusso, P.M. A phase II study of 17-allylamino-17-demethoxygeldanamycin in metastatic or locally advanced, unresectable breast cancer. *Breast Cancer Res Treat* **2012**, *131*, 933-937, doi:10.1007/s10549-011-1866-7.

62. Kaufmann, S.H.; Karp, J.E.; Litzow, M.R.; Mesa, R.A.; Hogan, W.; Steensma, D.P.; Flatten, K.S.; Loegering, D.A.; Schneider, P.A.; Peterson, K.L., et al. Phase I and pharmacological study of cytarabine and tanespimycin in relapsed and refractory acute leukemia. *Haematologica* **2011**, *96*, 1619-1626, doi:10.3324/haematol.2011.049551.
63. Rampal, R. HSP90 Inhibitor, AUY922, in Patients With Primary Myelofibrosis (PMF), Post-Polycythemia Vera Myelofibrosis (Post-PV MF), Post-Essential Thrombocythemia Myelofibrosis (Post-ET MF), and Refractory PV/ET. **2016**, https://www.clinicaltrials.gov/ct2/show/NCT01668173?term=AUY01668922+Rampal&draw=01668172&rank=01668171.
64. Speranza, G.; Anderson, L.; Chen, A.P.; Do, K.; Eugeni, M.; Weil, M.; Rubinstein, L.; Majerova, E.; Collins, J.; Horneffer, Y., et al. First-in-human study of the epichaperome inhibitor PU-H71: clinical results and metabolic profile. *Investigational new drugs* **2018**, *36*, 230-239, doi:10.1007/s10637-017-0495-3.
65. Hong, D.; Said, R.; Falchook, G.; Naing, A.; Moulder, S.; Tsimberidou, A.M.; Galluppi, G.; Dakappagari, N.; Storgard, C.; Kurzrock, R., et al. Phase I study of BIIB028, a selective heat shock protein 90 inhibitor, in patients with refractory metastatic or locally advanced solid tumors. *Clinical cancer research : an official journal of the American Association for Cancer Research* **2013**, *19*, 4824-4831, doi:10.1158/1078-0432.CCR-13-0477.
66. de Bono, J.S.; Kristeleit, R.; Tolcher, A.; Fong, P.; Pacey, S.; Karavasilis, V.; Mita, M.; Shaw, H.; Workman, P.; Kaye, S., et al. Phase I pharmacokinetic and pharmacodynamic study of LAQ824, a hydroxamate histone deacetylase inhibitor with a heat shock protein-90 inhibitory profile, in patients with advanced solid tumors. *Clinical cancer research : an official journal of the American Association for Cancer Research* **2008**, *14*, 6663-6673, doi:10.1158/1078-0432.CCR-08-0376.
67. Ramaswamy, B.; Fiskus, W.; Cohen, B.; Pellegrino, C.; Hershman, D.L.; Chuang, E.; Luu, T.; Somlo, G.; Goetz, M.; Swaby, R., et al. Phase I-II study of vorinostat plus paclitaxel and bevacizumab in metastatic breast cancer: evidence for vorinostat-induced tubulin acetylation and Hsp90 inhibition in vivo. *Breast Cancer Res Treat* **2012**, *132*, 1063-1072, doi:10.1007/s10549-011-1928-x.
68. Ramanathan, R.K.; Egorin, M.J.; Erlichman, C.; Remick, S.C.; Ramalingam, S.S.; Naret, C.; Holleran, J.L.; TenEyck, C.J.; Ivy, S.P.; Belani, C.P. Phase I pharmacokinetic and pharmacodynamic study of 17-dimethylaminoethylamino-17-demethoxygeldanamycin, an inhibitor of heat-shock protein 90, in patients with advanced solid tumors. *J Clin Oncol* **2010**, *28*, 1520-1526, doi:10.1200/JCO.2009.25.0415.
69. Oh, W.K.; Galsky, M.D.; Stadler, W.M.; Srinivas, S.; Chu, F.; Bubley, G.; Goddard, J.; Dunbar, J.; Ross, R.W. Multicenter phase II trial of the heat shock protein 90 inhibitor, retaspimycin hydrochloride (IPI-504), in patients with castration-resistant prostate cancer. *Urology* **2011**, *78*, 626-630, doi:10.1016/j.urology.2011.04.041.
70. Ramanathan, R.K.; Trump, D.L.; Eiseman, J.L.; Belani, C.P.; Agarwala, S.S.; Zuhowski, E.G.; Lan, J.; Potter, D.M.; Ivy, S.P.; Ramalingam, S., et al. Phase I pharmacokinetic-pharmacodynamic study of 17-(allylamino)-17-demethoxygeldanamycin (17AAG, NSC 330507), a novel inhibitor of heat shock protein 90, in patients with refractory advanced cancers. *Clinical cancer research : an official journal of the American Association for Cancer Research* **2005**, *11*, 3385-3391, doi:10.1158/1078-0432.CCR-04-2322.
71. Iyer, G.; Morris, M.J.; Rathkopf, D.; Slovin, S.F.; Steers, M.; Larson, S.M.; Schwartz, L.H.; Curley, T.; DeLaCruz, A.; Ye, Q., et al. A phase I trial of docetaxel and pulse-dose 17-allylamino-17-demethoxygeldanamycin in adult patients with solid tumors. *Cancer chemotherapy and pharmacology* **2012**, *69*, 1089-1097, doi:10.1007/s00280-011-1789-3.
72. Modi, S.; Stopeck, A.T.; Gordon, M.S.; Mendelson, D.; Solit, D.B.; Bagatell, R.; Ma, W.; Wheler, J.; Rosen, N.; Norton, L., et al. Combination of trastuzumab and tanespimycin (17-AAG, KOS-953) is safe and active in trastuzumab-refractory HER-2 overexpressing breast cancer: a phase I dose-escalation study. *J Clin Oncol* **2007**, *25*, 5410-5417, doi:10.1200/JCO.2007.11.7960.

73. Heath, E. 17-AAG in Treating Patients With Metastatic Prostate Cancer That Did Not Respond to Previous Hormone Therapy. **2017**, https://www.clinicaltrials.gov/ct2/show/NCT00118092?term=00118017-AAG+Heath&draw=00118092&rank=00118091.
74. Younes, A. 17-AAG in Treating Patients With Relapsed or Refractory Anaplastic Large Cell Lymphoma, Mantle Cell Lymphoma, or Hodgkin's Lymphoma. **2014**, https://www.clinicaltrials.gov/ct2/show/NCT00117988?term=00117917-AAG+Younes&draw=00117982&rank=00117981.
75. Kim, A.; Lu, Y.; Okuno, S.H.; Reinke, D.; Maertens, O.; Perentesis, J.; Basu, M.; Wolters, P.L.; De Raedt, T.; Chawla, S., et al. Targeting Refractory Sarcomas and Malignant Peripheral Nerve Sheath Tumors in a Phase I/II Study of Sirolimus in Combination with Ganetespib (SARC023). *Sarcoma* **2020**, *2020*, 5784876, doi:10.1155/2020/5784876.
76. Jhaveri, K.; Chandarlapaty, S.; Lake, D.; Gilewski, T.; Robson, M.; Goldfarb, S.; Drullinsky, P.; Sugarman, S.; Wasserheit-Leiblich, C.; Fasano, J. A phase II open-label study of ganetespib, a novel heat shock protein 90 inhibitor for patients with metastatic breast cancer. *Clinical breast cancer* **2014**, *14*, 154-160.
77. Hendrickson, A.E.; Oberg, A.L.; Glaser, G.; Camoriano, J.K.; Peethambaram, P.P.; Colon-Otero, G.; Erlichman, C.; Ivy, S.P.; Kaufmann, S.H.; Karnitz, L.M., et al. A phase II study of gemcitabine in combination with tanespimycin in advanced epithelial ovarian and primary peritoneal carcinoma. *Gynecol Oncol* **2012**, *124*, 210-215, doi:10.1016/j.ygyno.2011.10.002.
78. Linehan, W.M. 17AAG to Treat Kidney Tumors in Von Hippel-Lindau Disease. **2011**, https://www.clinicaltrials.gov/ct2/show/NCT00088374?term=00088317-AAG+linehan&draw=00088372&rank=00088371.
79. Subramaniam, D.S.; Liu, S.V.; Crawford, J.; Kramer, J.; Thompson, J.; Wang, H.; Giaccone, G. A Phase Ib/II Study of Ganetespib With Doxorubicin in Advanced Solid Tumors Including Relapsed-Refractory Small Cell Lung Cancer. *Front Oncol* **2018**, *8*, 64, doi:10.3389/fonc.2018.00064.
80. Corp., S.P. A Study Evaluating STA-9090 in Patients With Metastatic and/or Unresectable Gastrointestinal Stromal Tumor (GIST). **2016**, https://www.clinicaltrials.gov/ct2/show/NCT01039519?term=NCT01039519&draw=01039512&rank=01039511.
81. Banerji, U.; O'Donnell, A.; Scurr, M.; Pacey, S.; Stapleton, S.; Asad, Y.; Simmons, L.; Maloney, A.; Raynaud, F.; Campbell, M., et al. Phase I pharmacokinetic and pharmacodynamic study of 17-allylamino, 17-demethoxygeldanamycin in patients with advanced malignancies. *J Clin Oncol* **2005**, *23*, 4152-4161, doi:10.1200/JCO.2005.00.612.
82. Heath, E.I.; Hillman, D.W.; Vaishampayan, U.; Sheng, S.; Sarkar, F.; Harper, F.; Gaskins, M.; Pitot, H.C.; Tan, W.; Ivy, S.P., et al. A phase II trial of 17-allylamino-17-demethoxygeldanamycin in patients with hormone-refractory metastatic prostate cancer. *Clinical cancer research : an official journal of the American Association for Cancer Research* **2008**, *14*, 7940-7946, doi:10.1158/1078-0432.CCR-08-0221.
83. Solit, D.B.; Osman, I.; Polsky, D.; Panageas, K.S.; Daud, A.; Goydos, J.S.; Teitcher, J.; Wolchok, J.D.; Germino, F.J.; Krown, S.E., et al. Phase II trial of 17-allylamino-17-demethoxygeldanamycin in patients with metastatic melanoma. *Clinical cancer research : an official journal of the American Association for Cancer Research* **2008**, *14*, 8302-8307, doi:10.1158/1078-0432.CCR-08-1002.
84. Cercek, A. Clinical and Translational Study of STA-9090. **2015**, https://www.clinicaltrials.gov/ct2/show/study/NCT01111838?term=Cercek+ganetespib&draw=01111832&rank=01111831.
85. Vaishampayan, U.N.; Burger, A.M.; Sausville, E.A.; Heilbrun, L.K.; Li, J.; Horiba, M.N.; Egorin, M.J.; Ivy, P.; Pacey, S.; Lorusso, P.M. Safety, efficacy, pharmacokinetics, and pharmacodynamics of the combination of sorafenib and tanespimycin. *Clinical cancer research : an official journal of the American Association for Cancer Research* **2010**, *16*, 3795-3804, doi:10.1158/1078-0432.CCR-10-0503.

86. Ramalingam, S.S.; Egorin, M.J.; Ramanathan, R.K.; Remick, S.C.; Sikorski, R.P.; Lagattuta, T.F.; Chatta, G.S.; Friedland, D.M.; Stoller, R.G.; Potter, D.M., et al. A phase I study of 17-allylamino-17-demethoxygeldanamycin combined with paclitaxel in patients with advanced solid malignancies. *Clinical cancer research : an official journal of the American Association for Cancer Research* **2008**, *14*, 3456-3461, doi:10.1158/1078-0432.ccr-07-5088.
87. Orlemans, E. Safety and Efficacy of SNX-5422 in Human Epidermal Growth Factor Receptor 2 (HER2) Positive Cancers. **2015**, https://www.clinicaltrials.gov/ct2/show/results/NCT01848756?term=SNX-01845422&draw=01848752&rank=01848754.
88. Moley, J. A Phase II Trial of 17-Allylaminogeldanamycin (17AAG) in Advanced Medullary and Differentiated Thyroid Carcinoma. **2017**, https://www.clinicaltrials.gov/ct2/show/results/NCT00118248?term=Moley+aag&draw=00118242&rank=00118241.
89. Gartner, E. Tanespimycin in Treating Women With Refractory Locally Advanced or Metastatic Breast Cancer. **2017**, https://www.clinicaltrials.gov/ct2/show/study/NCT00096109?term=00096117-AAG+gartner&draw=00096102&rank=00096101.
90. Weigel, B.J.; Blaney, S.M.; Reid, J.M.; Safgren, S.L.; Bagatell, R.; Kersey, J.; Neglia, J.P.; Ivy, S.P.; Ingle, A.M.; Whitesell, L., et al. A phase I study of 17-allylaminogeldanamycin in relapsed/refractory pediatric patients with solid tumors: a Children's Oncology Group study. *Clinical cancer research : an official journal of the American Association for Cancer Research* **2007**, *13*, 1789-1793, doi:10.1158/1078-0432.CCR-06-2270.
